# End-of-life decision making for cancer patients in an intensive care unit

**DOI:** 10.1186/2197-425X-3-S1-A651

**Published:** 2015-10-01

**Authors:** M Tavares, I Neves, F Coelho, O Afonso, A Martins, F Faria

**Affiliations:** IPO - Porto, Porto, Portugal

## Introduction

Patients with advanced malignances are at a high risk of developing complications that lead to an Intensive Care Unit (ICU) admission. Despite improvements in ICU-level care, mortality rates for some patients remain especially high. Limitation of therapy is an integral component of high-quality care of cancer patients in the ICU.

## Objectives

Describe the practice and analyze associated factors of life-sustaining treatment in the 8-bed ICU of a cancer specialized center.

## Methods

Retrospective surveillance of adult patients (aged more than 18 years) admitted to the ICU from January/2010 to December/2014. For patients with more than one admission, only the last one was analyzed. Patients were divided into two groups: withdrawing or withholding life support (WWLS), and full life support, as suggested in the literature. Predictive factors of WWLS were identified using multivariate logistic regression analysis.

## Results

Among 1511 patients admitted to ICU, 1309 (86,6%) had solid tumors and 202 (13,4%) had some kind of hematological malignancy. A small group had received stem-cell transplant (4,9%). The median age was 62 (18, minimum and 90, maximum) years and 58% were male. Thirteen percent (196/1511) of patients had limitation of therapy (WWLS). We observed no difference in the annual prevalence during the study period (p=0,631). Primary reasons for the decision concerned malignancy status namely refractoriness to therapy and progressive disease. Hospital mortality was 39% (590/1511) and 33,2% of deaths occurred after WWLS. WWLS was independently associated with age, surgical status, length of mechanical ventilation, length of stay, APACHE score and organ failure (table 1).

**Table 1:** Multivariate logistic regression analysis.

Variable	Odds Ratio	95% CI	p
Age	1,020	0,899-2,533	0,008
Surgical status	0,257	0,137-0,483	< 0,001
Duration of mechanical ventilation	1,004	1,002-1,006	0,001
Length of stay in the ICU	0,948	0,902-0,996	0,036
APACHE ≥ 35	4,327	2,789-6,713	< 0,001
MODS	3,926	2,569-6,001	< 0,001

## Conclusions

End-of-life practice has been a routine in our center during the last 5 years (13% of admissions). As demonstrated previously in general ICU, clinical parameters seem to be major determinants of WWLS decisions in cancer patients. Consensus statements may help physicians in the difficult task of end-of-life decision making.

## References

[1] Azoulay E (2009). End-of-life practices in 282 intensive care units: data from the SAPS 3 database. Intensive Care Med.

[2] Sprung CL (2003). End-of-life practices in European intensive care units: the Ethicus Study. JAMA.

[3] Truog RD (2008). Recommendations for end-of-life care in the intensive care unit: A consensus statement by the American College of Critical Care Medicine. Crit Care Med.

